# Editorial Notes, News and Miscellany

**Published:** 1912-10

**Authors:** 


### Editorial Notes, News and Miscellany.

Dr. W. A. King, President Bexar County Medical Association, has been appointed a member of the San Antonio City Board of Health, vice Dr. T. T. Jackson, Surgeon S. P. Railroad System, who resigns because of his appointment to this last position.

---

President Taft, with the confirmation of the Senate, on July 13th, commissioned William Edward Fitch, M. D. (Editor of *Pediatrics,* New York City), to be a first lieutenant in the Medical Reserve Corps of the Army of the United States, with rank dating from July 3, 1912.

---

"Proserpine" Again.—Readers will recall the spicy letters a few years ago signed "Proserpine." We have another of the same kind from her, to appear in next issue, criticising a paper in our

June number on "How to Keep Our Husbands, by a Wife and Mother." Proserpine is Elois Shepherd, of Houston, a well known writer.

---

THE FIRST NORTHER.—

Now fades the glimmering straw hat out of sight,
And all the clothes a wintry aspect wear,—
Save where the editor,—always in a tight,—
Must wear his linen duster still,—or go bare!

Please remit!

---

SOME COMMON ERRORS OF SPEECH.—Don't say "enthuse." Don't say "those kind." Don't say "feeling badly." Don't say "quite a few." Don't say "none were hurt." Don't say "between you and I." Don't say "a widow woman." Don't say "six aught six." Don't say "it don't do." Don't say "consider," when you mean "think" or "deem."

---

LAYTON, UTAH, September 16, 1912.

*Dr. F. E. Daniel, Editor Texas Medical Journal, Austin, Texas.*

MY DEAR DOCTOR: I congratulate you and your wife on the establishment of "The Woman's Department." I intend to refer to and quote from it in the near future.

DR. FREDERIC CLIFT,
Editor *Utah Medical Journal.*

---

THE MEDICAL ASSOCIATION OF THE SOUTHWEST will meet at Hot Springs, Ark., October 8, 9, 10 (inst.). The Secretary, Dr. F. H. Clark, has sent us the program, but I have no room for it this issue. I note that Prof. M. L. Graves, M. D., of the University Medical College, Galveston, will deliver the "Oration in Medicine," and that papers will be read by the following named Texas physicians: Dr. Joe Becton, Greenville; Dr. K. H. Beall, of Fort Worth; Dr. E. C. Eberly, Dr. Jno. S. Turner (President Texas State Medical Association), Dr. E. H. Cary, Dr. J. O. McReynolds, all of Dallas; Dr. W. W. Moore, of Paris, and Dr. H. L. Hilgartner, of Austin.

---

CONSISTENCY A JEWEL.—The following are some of the beautiful inconsistencies of our social system:

We permit the principal factor in crime, insanity, prostitution and pauperism, domestic infelicity and divorce—the infernal "saloon"—to flourish in rank luxuriance, then hang or imprison and torture its criminal victims; we build palaces—a million dollars a year—for the care of the insane, who are permitted to propagate their kind. Seven men were slaughtered at Sing Sing recently by law, and nine are to be killed this month at San Quintin, California. We bury a dead body to get rid of it, but embalm and preserve it from decay. We permit the pollution of our water supply, and build million-dollar filters to "purify" the water. We permit flies to breed by the million—then try to swat them out of existence. Oh, what's the use? We are "civilized" all right.

